# Estriol and Progesterone: A New Role for Sex Hormones

**Published:** 2006-12

**Authors:** Sanja Draca

**Affiliations:** *Clinic “Dr M.Zotović”, Belgrade, Serbia*

**Keywords:** estriol, progesterone, autoimmune diseases, traumatic brain injury

## Abstract

The physiological states pregnancy and parturition are undoubtedly associated with clinical changes of most of the autoimmune diseases. An altered Th1/Th2 balance has been proposed as an underlying mechanism. A pregnancy has protective effect on Th1-mediated autoimmune diseases, and a deteriorative effect on Th2-mediated autoimmune diseases. Numerous studies, both experimental and clinical, imply that estriol and progesterone, at high doses (such as those achieved during pregnancy), have potent anti-inflammatory and neuroprotective roles. These studies, as well as a further investigation of pregnancy-associated immunomodulation, can be used to advance and develop a new therapy approaches.

## INTRODUCTION

It has been increasingly apparent that effects of sex hormones extend far beyond their predominant role in sexual differentiation and reproduction. Sex hormones appear to be involved in regulating the immune response, which is evident from a striking difference between a male and female immune response, and the alteration of female immune response during pregnancy, lactation and different phases of menstrual cycle (1). There is a distinct female preponderance of most autoimmune diseases, as well as a gender dimorphism related to a disease progression of autoimmune diseases. Moreover, numerous *in vivo* studies reported that course and severity of certain autoimmune diseases are modulated not only by castration or sex steroid administration, but also by pregnancy and lactation (2).

Immune response is regulated by components of innate immunity, as well as by components of acquired immunity, such as T helper (Th) cells subdivided to Th1 and Th2 subsets. A third Th cell subset, a regulatory T cell (Treg), has been recently identified. Th1 cells primarily secrete pro-inflammatory cytokines as IFN (interferon)-γ, IL (interleukin)-2 and TNF (tumor necrosis factor)-α, which promote cellular immunity, whereas Th2 cells secrete a different set of anti-inflammatory cytokines IL-4, IL-5, IL-6 and IL-13, which promote humoral immunity. Treg cells produce cytokines like IL-4, IL-10 and TGF (transforming growth factor)-β, and have suppressive properties for both Th1 and Th2 cells. A Th1 and Th2 responses are mutually inhibitory, and to some extent opposing, whereas Treg prevent the derangement of the Th1/Th2 balance in either direction (3, 4).

## ESTRIOL AND PROGESTERONE: IMMUNITY AND NEUROPROTECTION

Copious data support the concept of an altered Th1/Th2 balance during pregnancy, suggesting that pregnancy can be regarded as a Th2-type phenomenon. A dominance of Th-2 type cells prevents a rejection of antigenically foreign fetus by a cell-mediated immune attack (5). Pregnancy-associated Th2 shift has been proposed as a mechanism underlying the improvement of Th1-mediated autoimmune diseases (as rheumatoid arthritis, multiple sclerosis (MS), autoimmune thyoriditis, uveitis, and psoriatic arthritis), or a deterioration of Th2-mediated autoimmune diseases (as systemic lupus erythematosus).

A remarkable feature of pregnancy is a successful adaptation of woman to enormous endocrine changes as increased production of estrogens, progesterone (PRG), corticosteroids, and numerous protein hormones (some are placental specific and unique to pregnancy) (6). During pregnancy PRG serum level increases by a factor of 4, while estrogen estriol (E3) serum concentration increases by a factor of 20. Normally, E3 is not measurable in serum. E3 is produced principally from 16α-hydroxydehydroepia ndrosterone sulfate in the fetal plasma. Its level rises during pregnancy, peaking in the third trimester, and drops postpartum. In the urine of nonpregnant women the ratio of E3 to E1 (estron) plus E2 (estradiol) is approximately 1:1, whereas in the urine of pregnant women the ratio is 10:1 or more. In pregnant women at or near term there is a daily production of about 300 μmol (80 mg) of E3 and 1 mmol (300 mg) of PRG (6).

Estrogens and PRG induce a substantial Th2 shift, most likely at concentrations associated with pregnancy.

The estrogens are known to exert opposing, bimodal, dose-specific effect to immune response. Low levels facilitate a cell-mediated pro-inflammatory immune response, whereas their relatively high levels, such as those achieved during pregnancy, promote an anti-inflammatory Th2 response (7). Studies in the last decade showed that estrogens in high concentrations have a beneficial effect on the experimental autoimmune encephalomyelitis (EAE), a mouse model of chronic relapsing-remitting form of human MS. In a recent EAE study, a long-term treatment of castrated female mice with E3, given at doses corresponding to those seen during late stage of pregnancy, delayed the disease onset even for a longer time than treatment with E2 (8). Autoantigen myelin basic protein (MBP)-specific T-lymphocyte responses from E3-treated EAE mice are characterized by significantly increased production of cytokine IL-10 (9). Moreover, it has been reported that estriol’s ameliorative effect is not gender-specific. E3 treatment decreased the EAE disease severity, as well as pro-inflammatory cytokine production in both females and males (10). Furthermore, in a pilot trial, oral E3 treatment of women with relapsing-remitting MS (RRMS) caused significant decreases in the number of gadolinium-enhancing lesions on brain magnetic resonance imaging (MRI), and significant increases of IL-5 and IL-10 levels. These changes in cytokines correlated with reductions of enhancing lesions on brain MRI (11, 12). In women with secondary progressive MS (SPMS) a lack of treatment effect is believed to support a concept of different immune dysregulation.

Estrogens can display a wide range of neuroprotective activities, including stabilization of neurotransmission, inhibition of apoptosis, and direct antioxidant activities which may be important for defense against oxidative stress (13). Similarly, physiological concentrations of PRG and estrogens, consistent with late pregnancy, are shown to inhibit activated microglia, resident central nervous system (CNS) cells associated with a wide variety of neuroimmunological diseases, including MS. In addition, PRG and estrogens inhibited microglial production of NO (nitric oxide) and TNFα, molecules that can be toxic to myelin-producing oligodendrocytes and neurons (14, 15).

PRG mediates promotion of Th2 response, inducing conversion of Th0 cells into Th2 cells. At concentrations comparable to those present at the maternal-fetal interface PRG induces a development of Ag-specific CD4+ T cell lines that show enhanced ability to produce IL-4 and IL-5 (16). Also, PRG directly suppresses T cell differentiation into Th1 cells, and enhances IL-10-producing Th2 cells (17). On the other hand, PRG has been shown to provide substantial neuroprotective effect in male and female animals after brain or spinal cord injury. As an acute post-injury treatment PRG reduces cerebral edema, neuronal loss, and inflammation process (18-20). In a recent pilot clinical trial using PRG to treat traumatic brain injury (TBI) patients, stable PRG concentrations were rapidly achieved following continuous intravenous infusion, whereas alterations in PRG pharmacokinetics were not gender specific. Also, PRG showed possible signs of benefit with no appreciable effects on heart rate, coagulopathy or infection rate, important considerations in seriously injured patients (20, 21).

## CONCLUSION

Taken together, the above studies imply that the effects of sex hormones on the immune and nervous system are clearly apparent, although imperfectly understood (Table 1). The transition from animal and cellular models to human therapies is fraught with difficulty, especially concerning some large clinical trials suggesting that some hormone therapies may increase the risk of several diseases, including stroke. The results of these studies may be due to the doses, specific hormone used, and sub-population of patients. However, a several clinical trials on the effects of sex hormones to MS or TBI have been performed. Their results suggest that hormone therapy may represent a new therapeutic tool to combat certain diseases. Estriol, the safest of three estrogens, is a candidate for treatment women with RRMS, based on a recent phase I clinical trial. Since estriol’s ameliorative effect is not gender-specific, the possibility of estriol treatment in men exists. In a pilot trial, PRG has already been used in both sexes of patients with TBI.

**Table 1:** A summary of experimental and clinical studies on the effects of pregnancy-related sex hormones

Langer-Gould A, *et al*. Late pregnancy suppresses relapses in experimental autoimmune encephalomyelitis: evidence for a suppressive pregnancy-related serum factor. *J. Immunol* 2002; 169: 1084. Jansson L, *et al*. Estrogen induces a potent suppression of experimental autoimmune encephalomyelitis and collagen-induced arthritis in mice. *J. Neuroimmunol* 1994; 53: 203. Kim S, *et al*. Estriol ameliorates autoimmune demyelinating disease: implications for multiple sclerosis. *Neurology* 1999; 52: 1230. Soldan S, *et al*. Immune modulation in multiple sclerosis patients treated with the pregnancy hormone estriol. *J. Immunol* 2003; 171: 6267. Sicote N, *et al*. Treatment of multiple sclerosis with the pregnancy hormone estriol. *Ann. Neurol* 2002; 52: 421. Thomas AJ, *et al*. Progesterone is neuroprotective after acute experimental spinal cord trauma in rats. *Spine* 1999; 24: 2134. Roof RL, *et al*. Progesterone rapidly decreases brain edema: treatment delayed up to 24 hours is still effective. *Exp. Neurol* 1996; 138: 246. Wright DW, *et al*. Steady-state serum concentrations of progesterone following continuous intravenous infusion in patients with acute moderate to severe traumatic brain injury. *J. Clin. Pharmacol* 2005; 45: 640.

Thus, further randomized clinical trials are necessary. It is important to minimize the risk of treatment, and design therapies optimized for anti-inflammatory and neuroprotective efficacy.
